# Supplementary material for: sCellST predicts single-cell gene expression from H& E images
Source: Nat Commun. 2026 Jan 9;17:1194. doi: 10.1038/s41467-025-67965-1 (PMC12858858; doi:10.1038/s41467-025-67965-1)
Supplement: Supplementary file 1 — Supplementary Information [file 41467_2025_67965_MOESM1_ESM.pdf]

Supplementary material for: "sCellST Predicts Single-Cell Gene Expression from H&E Images"

Loïc Chadoutaud[1, 2, 3], Marvin Lerousseau[1, 2, 3, 4], Daniel Herrero-Saboya[1, 2, 3, 5], Julian Ostermaier[1, 2, 3], Jacqueline Fontugne[6, 7, 8], Emmanuel Barillot[1, 2, 3, *], and Thomas Walter[1, 2, 3, *]

[1]Centre for Computational Biology (CBIO), Mines Paris, PSL University, 75006 Paris, France
[2]Institut Curie, 75248 Paris Cedex, France
[3]INSERM, U1331, 75248 Paris Cedex, France
[4]Present address: Spotlight Medical, Paris, France
[5]Computational Medicine, Servier Research & Development, Saclay, France
[6]Institut Curie, Department of Pathology, Saint-Cloud, France
[7]Institut Curie, CNRS, UMR144, Equipe labellisée Ligue Contre le Cancer, PSL Research University, Paris, France
[8]Université Paris-Saclay, UVSQ, Montigny-le-Bretonneux, France
[*]Corresponding authors: emmanuel.barillot@curie.fr, thomas.walter@minesparis.psl.eu

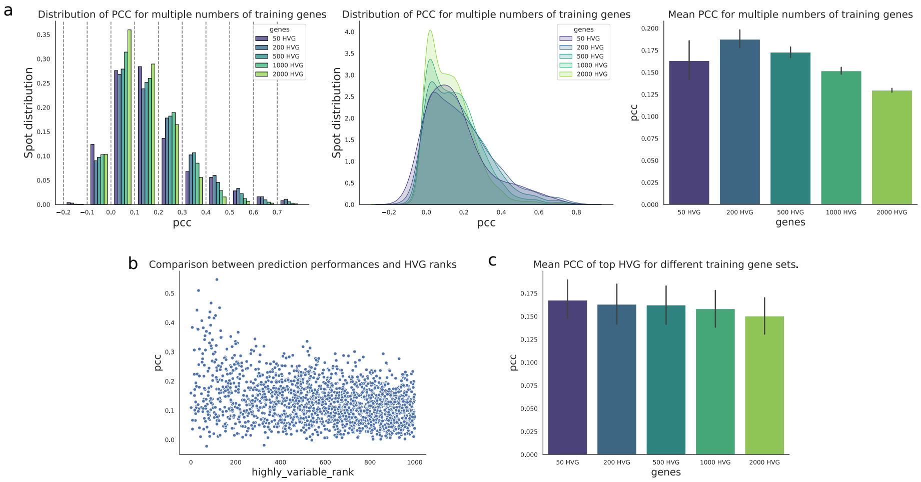

Supplementary Figure 1: **Impact of numbers of highly variable genes (HVG) on performances measured with Pearson correlation coefficient (PCC).** On a subset of the benchmarking study (Prostate dataset with leave-one-out strategy), we examined how model performance depends on the number of genes used for training. In (a), binned histograms and density plots show that increasing the number of genes does not necessarily add more difficult-to-predict genes; for example, the highest average PCC is achieved using 200 HVG. In (b), we demonstrate that the rank based on gene variability is a poor predictor of gene predictability. Finally, in (c), we present the mean PCC for the commons HVGs (n=48), showing that increasing the total number of genes reduces the model's predictive power on this subset. Source data are provided as a Source Data file.

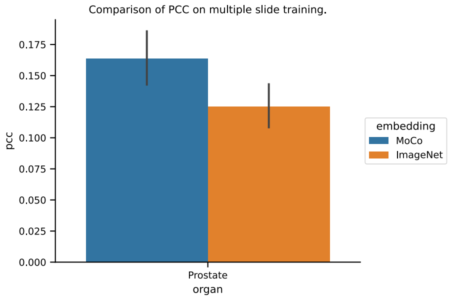

Supplementary Figure 2: **Impact of embedding quality on performances.** On a subset of the benchmarking study (Prostate dataset with leave-one-out strategy), we examined how model performances change with different embedding quality. We show that embedding obtained with MoCo are much better than the ones obtained from Transfer Learning. Each bar plot represents the mean Pearson correlation coefficient (error bars correspond to the 95% confidence interval obtained via bootstrapping) with n=50 genes and n=5 test slides. Source data are provided as a Source Data file.

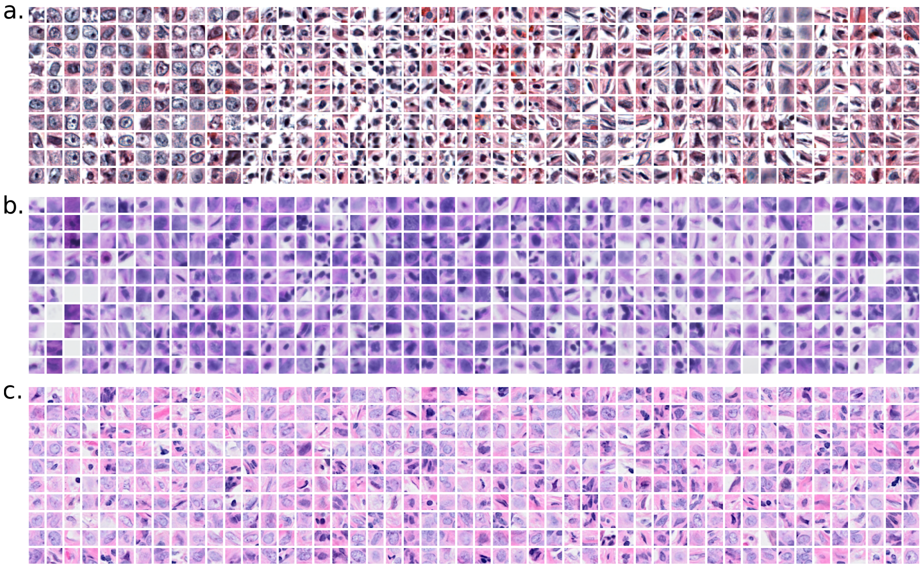

Supplementary Figure 3: Image galleries of randomly picked cells from the three different slide used in the Xenium experiments **(a)** Visium training slide TENX39, **(b)** Xenium NCBI785 and **(c)** Xenium TENX95. Each image corresponds to 12μm × 12μm.

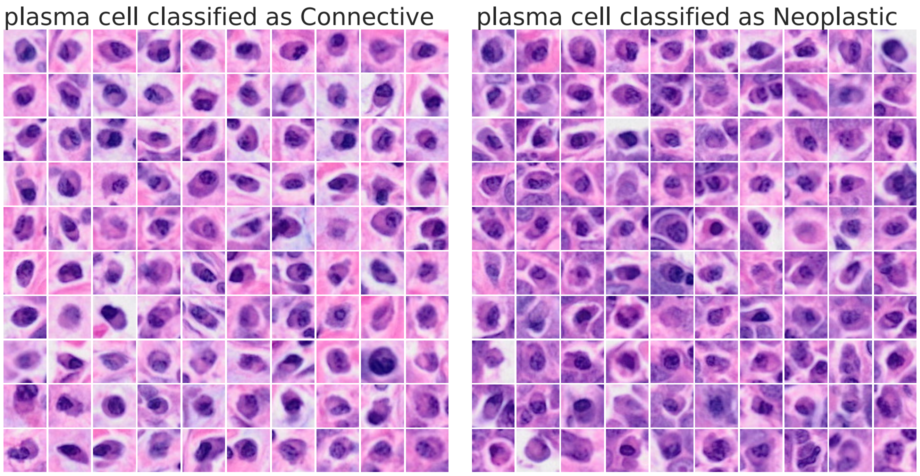

Supplementary Figure 4: Image galleries showing the highest-scoring images for the plasma cell score classified by CellViT as either neoplastic or connective. Each image corresponds to 13.1μm $\times$ 13.1μm

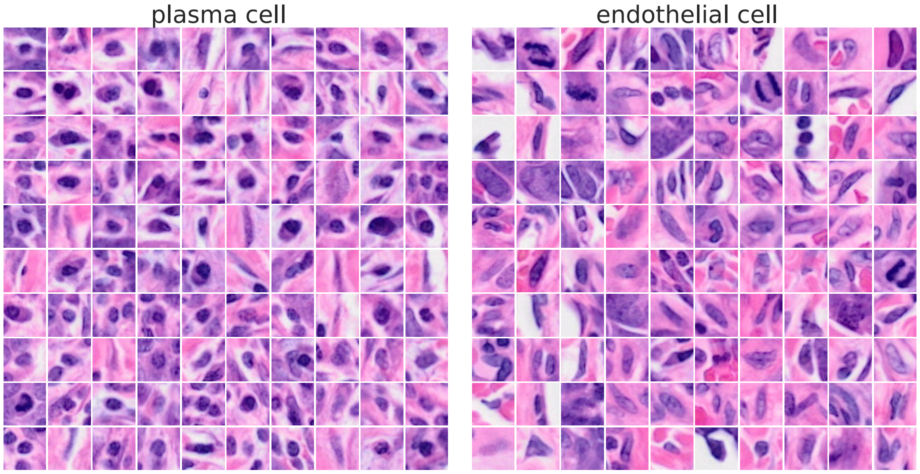

Supplementary Figure 5: Image galleries showing the highest-scoring images for the plasma cell score and endothelial cell score when sCellST is trained with ImageNet-derived-embeddings as input. Each image corresponds to 13.1μm $\times$ 13.1μm

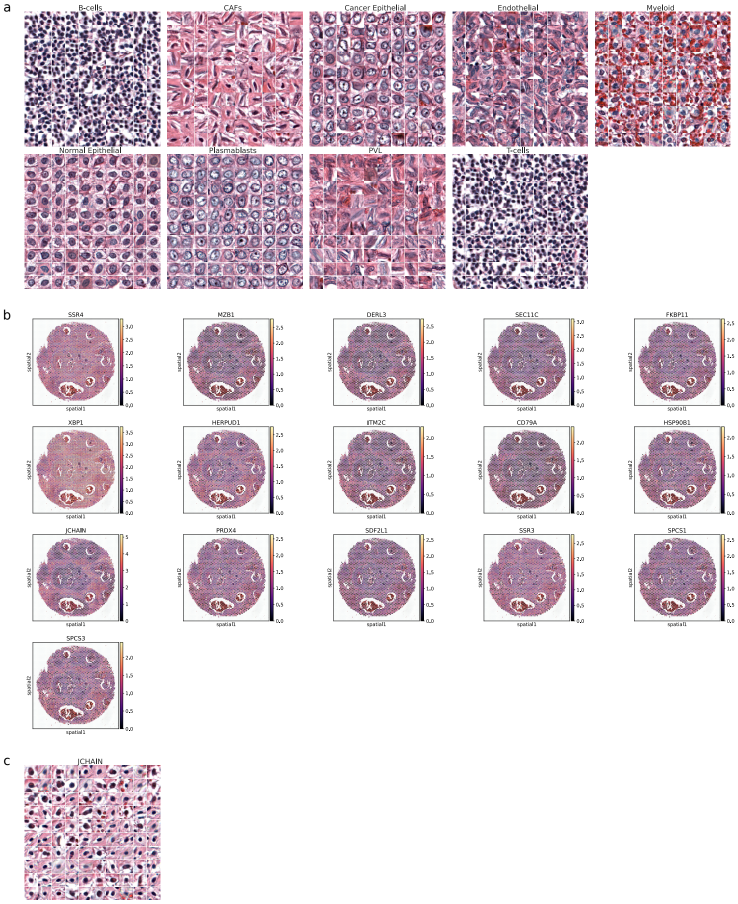

Supplementary Figure 6: **Galleries from breast cancer slide.** We computed cell type scores using cell images from a Visium breast cancer slide and generated galleries displaying the top 100 cells for each cell type in **a**. Galleries corresponding to B and T cells exhibit morphological features characteristic of lymphocytes. The gallery for myeloid cells, marked by genes associated with monocytes, also appears plausible and is supported by the presence of hematocytes in the images. Endothelial cells and perivascular-like cells (PVL) both display elongated nuclei, a trait also observed in cancer-associated fibroblasts. The distinction between cancerous and normal epithelial cells is particularly interesting, as cancer cells tend to appear larger and exhibit more prominent nucleoli. Finally, the plasmablast group appears less convincing, as the selected cells morphologically resemble cancer cells. Each image corresponds to 16.3μm $\times$ 16.3μm. However, as shown in **b**, the gene score distribution on the Visium slide indicates that most genes in the plasmablast marker set contribute minimal signal and are largely noisy. Notably, when focusing on JCHAIN, the most prominently expressed gene in the set, we recover the expected plasmablast-like morphology, as illustrated in **c**.

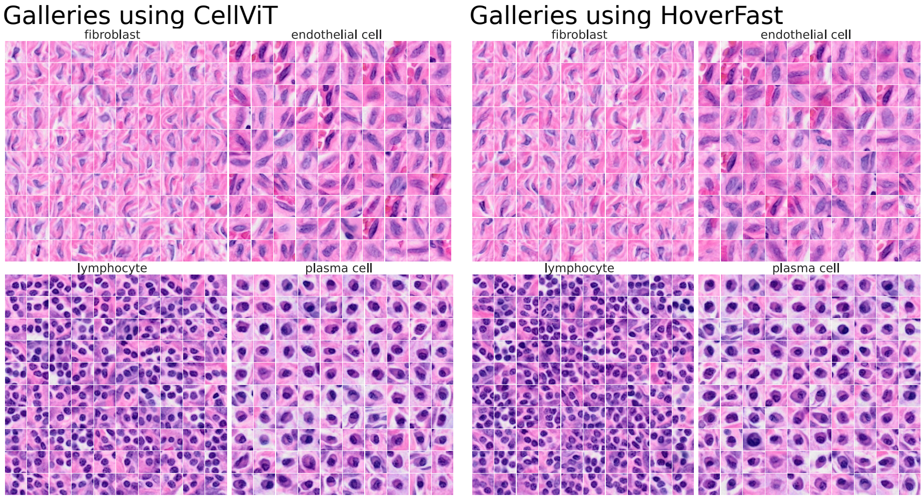

Supplementary Figure 7: **Impact of cell segmentation algorithm.** We replicated the analysis performed on the ovarian dataset in which we plotted galleries of cells based on cell type scores. We show the galleries obtained using CellViT on the left and using HoverFast on the right. As can be seen with these images, the similar patterns have been identified by sCellST regardless of the segmentation network used. Each image corresponds to $13.1\mu m \times 13.1\mu m$

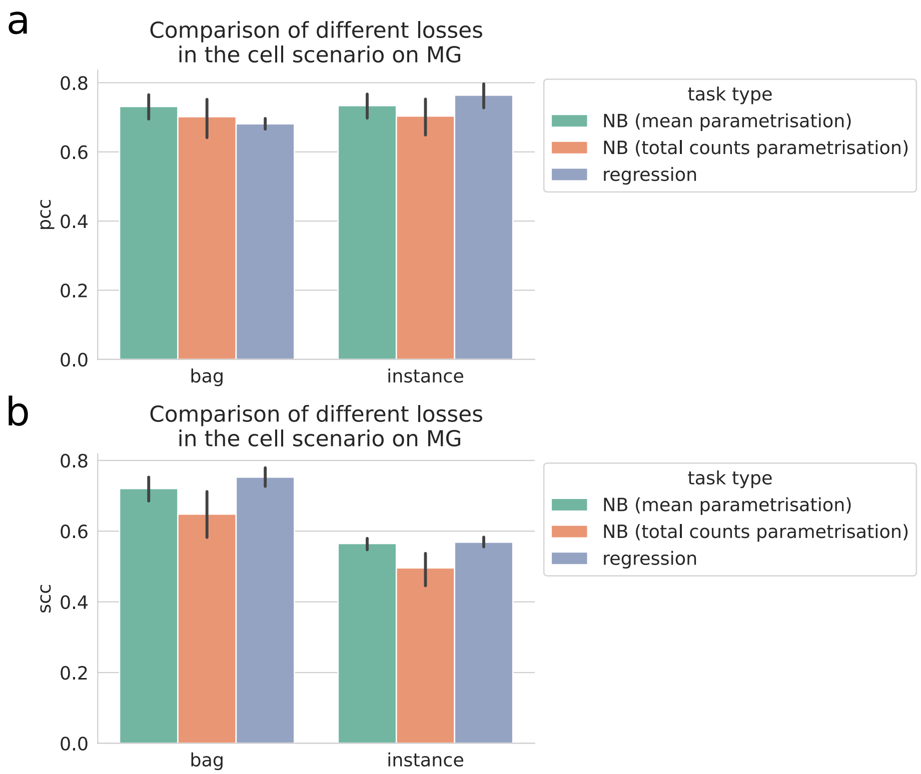

Supplementary Figure 8: Comparison of mean **(a)** Pearson correlation and **(b)** Spearman correlation (error bars correspond to the 95% confidence interval obtained via bootstrapping) in the cell scenario for marker genes (MG) n=51, at both the instance and bag levels when using different parametrisations of the loss function: classic regression, maximum likelihood with a negative binomial parametrised by the mean parameter, and maximum likelihood with a negative binomial parametrised by the total count parameter. Source data are provided as a Source Data file.

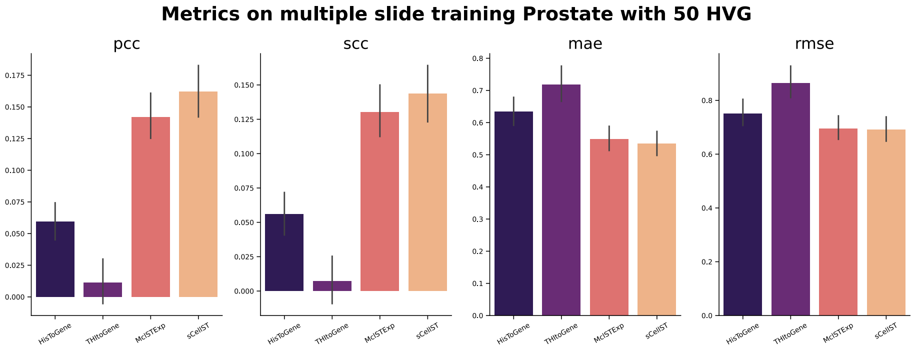

Supplementary Figure 9: **Impact of the choice of metrics** On a subset of the benchmarking study (Prostate dataset with leave-one-out strategy), we examined how model performances change with different metrics. All conclusion which can be drawn from correlation metrics are consistent with other metrics such as root Mean Squared Error (rMSE) and Mean Absolute Error (MAE). Each bar plot represents the mean Pearson correlation coefficient (error bars correspond to the 95% confidence interval obtained via bootstrapping) with n=50 genes and n=5 test slides. Source data are provided as a Source Data file.

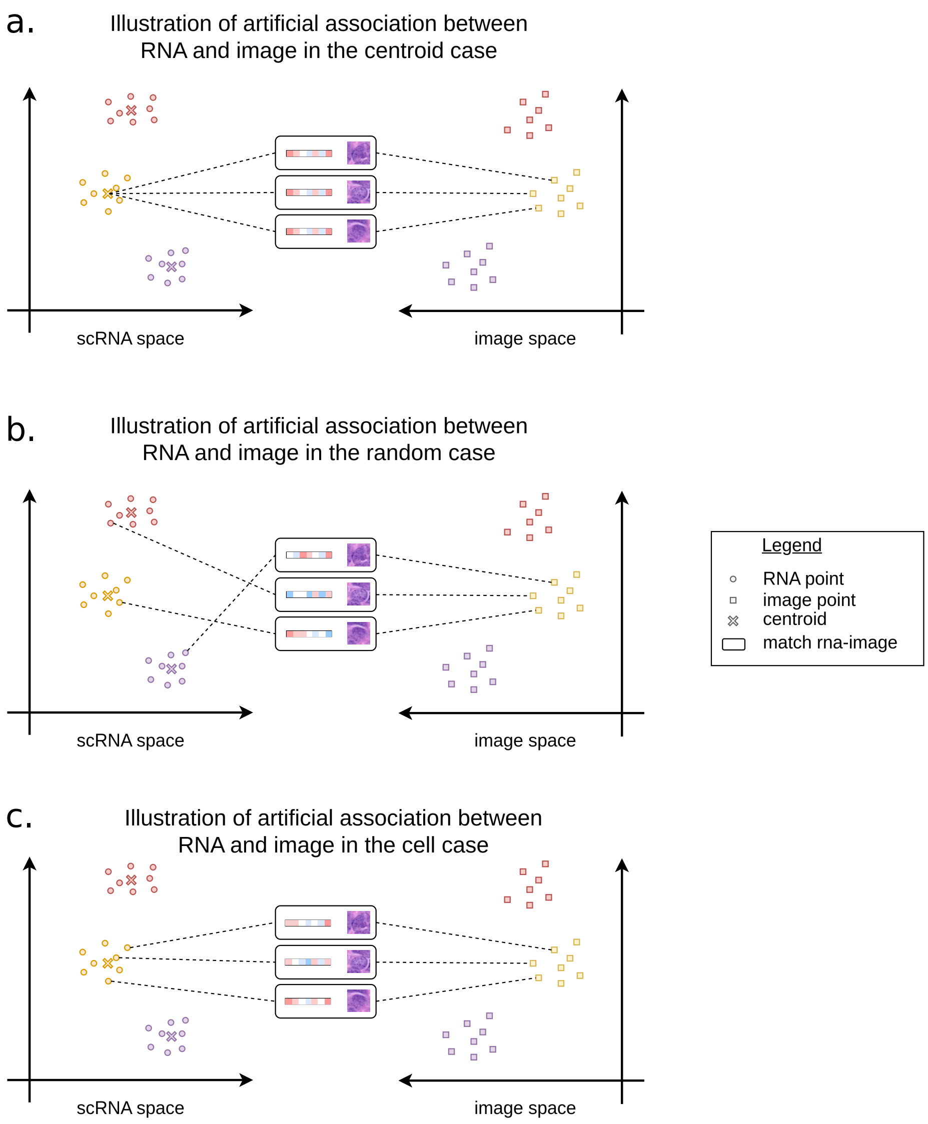

Supplementary Figure 10: Simulation framework illustrating cell image and gene expression (GE) attribution under **(a)** the centroid scenario, **(b)** the random scenario and **(c)** the cell scenario.

Cell type	Ovarian cancer slide	Breast cancer slide
Connective	68685	19470
Dead	105	30
Epithelial	60	15
Inflammatory	19618	16607
Neoplastic	221455	14688
**Total**	309923	50810

Supplementary Table 1: Number of cell per class detected by CellViT in H&E slides

Slide id	Number of genes
NCBI783	93
NCBI784	101
NCBI785	101
TENX94	93
TENX95	93
TENX96	126
TENX97	126
TENX98	93
TENX99	93

Supplementary Table 2: Number of SVG from the TENX39 breast cancer slides found in Xenium slides.

cell type	Top 20 genes
endothelial cell	A2M, ADGRL4, APP, CD34, CD93, CDH5, CLEC14A, COL4A1, COL4A2, EGFL7, ENG, GNG11, HSPG2, IGFBP7, INSR, PECAM1, RAMP2, SPARCL1, SPTBN1, VWF
fallopian tube secretory epithelial cell	BCAM, CD24, CD9, CLDN3, DSP, ELF3, EPCAM, FOLR1, KRT18, KRT19, KRT7, KRT8, MAL2, MSLN, MUC1, RPL8, S100A13, SLPI, SPINT2, WFDC2
fibroblast	AEBP1, C1R, C1S, CALD1, COL1A1, COL1A2, COL3A1, COL5A1, COL5A2, COL6A1, COL6A2, COL6A3, CTHRC1, DCN, LGALS1, LUM, MMP2, PCOLCE, RARRES2, SPARC
lymphocyte	ACAP1, BTG1, CCL5, CD2, CD3D, CD3E, CD3G, CD52, CD69, CORO1A, CXCR4, ETS1, EVL, GZMA, HCST, IL32, NKG7, PTPRC, TRAC, TSC22D3
plasma cell	CD79A, DERL3, FCRL5, FKBP11, FKBP2, HERPUD1, IGHG1, IGHG3, IGHG4, IGKC, JCHAIN, MZB1, PIM2, PRDX4, SEC11C, SPCS3, SSR4, TENT5C, TNFRSF17, XBP1

Supplementary Table 3: Marker genes extracted from the ovarian scRNA-seq dataset.

cell type	Top 20 genes
B-cells	BANK1, BTG1, CD37, CD52, CD74, CD79A, CD79B, CD83, FAU, HLA-DPA1, HLA-DPB1, HLA-DQA1, HLA-DQB1, HLA-DRA, HLA-DRB1, HLA-DRB5, IRF8, LAPTM5, MS4A1, VPREB3
CAFs	C1S, CALD1, COL1A1, COL1A2, COL3A1, COL6A1, COL6A2, COL6A3, CTSK, DCN, ISLR, LGALS1, LUM, MXRA8, PCOLCE, RARRES2, SERPINF1, SERPING1, SFRP2, SPARC
Cancer Epithelial	AZGP1, CD24, CLDN3, CLDN4, CLDN7, CYB5A, DSTN, ELF3, EPCAM, FXYD3, KRT18, KRT19, KRT7, KRT8, MGST1, PERP, RAB25, SMIM22, SPINT2, TSTD1
Endothelial	A2M, ADGRL4, AQP1, CLEC14A, EGFL7, EMCN, ENG, ESAM, GNG11, HSPG2, IFI27, IFITM3, IGFBP7, NPDC1, PECAM1, PLVAP, RAMP2, RNASE1, SPARCL1, VWF
Myeloid	AIF1, C1QA, C1QB, CD68, CD74, CST3, CTSB, FCER1G, FTL, GPX1, HLA-DPA1, HLA-DPB1, HLA-DRA, HLA-DRB1, LYZ, MS4A6A, NPC2, PSAP, SPI1, TYROBP
Normal Epithelial	APP, ATF3, AZGP1, CD9, CLDN4, CLU, CRYAB, KRT15, KRT18, KRT7, KRT8, MAFF, MGP, PERP, SFRP1, SPINT2, TACSTD2, TM4SF1, TPM1, WFDC2
PVL	ACTA2, ADIRF, BGN, CALD1, CAV1, IFITM3, IGFBP7, LHFPL6, MCAM, MFGE8, MYL9, NDUFA4L2, NOTCH3, PLAC9, SOD3, SPARC, SPARCL1, TAGLN, TPM1, TPM2
Plasmablasts	CD79A, DERL3, FKBP11, FKBP2, HERPUD1, HSP90B1, IGLL5, ITM2C, JCHAIN, MZB1, PRDX4, SDF2L1, SEC11C, SEC61B, SPCS1, SPCS2, SPCS3, SSR3, SSR4, XBP1
T-cells	ARHGDIB, B2M, BTG1, CCL5, CD2, CD3D, CD3E, CD52, CD69, CD7, CXCR4, CYTIP, IL32, IL7R, PTPRC, RAC2, SRGN, TMSB4X, TNFAIP3, TSC22D3

Supplementary Table 4: Marker genes extracted from the breast scRNA-seq dataset.
