# Supplementary material for: sCellST predicts single-cell gene expression from H& E images
Source: Nat Commun. 2026 Jan 9;17:1194. doi: 10.1038/s41467-025-67965-1 (PMC12858858; doi:10.1038/s41467-025-67965-1)
Supplement: Supplementary file 2 — Description of Additional Supplementary Files [file 41467_2025_67965_MOESM2_ESM.pdf]

Description of Additional Supplementary Files:

Supplementary Data 1: Pearson correlation for genes shared betweenthe top 1000 spatially variable genes (SVGs) identified in a Visium breast cancer slide and Xenium slides evaluated across multiple Xenium breast cancer slides
