# Supplementary material for: sCellST predicts single-cell gene expression from H& E images
Source: Nat Commun. 2026 Jan 9;17:1194. doi: 10.1038/s41467-025-67965-1 (PMC12858858; doi:10.1038/s41467-025-67965-1)
Supplement: Supplementary file 4 — Reporting Summary [file 41467_2025_67965_MOESM4_ESM.pdf]

nature portfolio

Corresponding author(s): Thomas Walter

Last updated by author(s): 2025/10/29

Reporting Summary

Nature Portfolio wishes to improve the reproducibility of the work that we publish. This form provides structure for consistency and transparency in reporting. For further information on Nature Portfolio policies, see our Editorial Policies and the Editorial Policy Checklist.

Statistics

For all statistical analyses, confirm that the following items are present in the figure legend, table legend, main text, or Methods section.

n/a	Confirmed	
[ ]	[x]	The exact sample size (*n*) for each experimental group/condition, given as a discrete number and unit of measurement
[x]	[ ]	A statement on whether measurements were taken from distinct samples or whether the same sample was measured repeatedly
[ ]	[x]	The statistical test(s) used AND whether they are one- or two-sided<br>*Only common tests should be described solely by name; describe more complex techniques in the Methods section.*
[x]	[ ]	A description of all covariates tested
[ ]	[x]	A description of any assumptions or corrections, such as tests of normality and adjustment for multiple comparisons
[ ]	[x]	A full description of the statistical parameters including central tendency (e.g. means) or other basic estimates (e.g. regression coefficient) AND variation (e.g. standard deviation) or associated estimates of uncertainty (e.g. confidence intervals)
[x]	[ ]	For null hypothesis testing, the test statistic (e.g. *F*, *t*, *r*) with confidence intervals, effect sizes, degrees of freedom and *P* value noted<br>*Give P values as exact values whenever suitable.*
[x]	[ ]	For Bayesian analysis, information on the choice of priors and Markov chain Monte Carlo settings
[x]	[ ]	For hierarchical and complex designs, identification of the appropriate level for tests and full reporting of outcomes
[ ]	[x]	Estimates of effect sizes (e.g. Cohen's *d*, Pearson's *r*), indicating how they were calculated

Our web collection on statistics for biologists contains articles on many of the points above.

Software and code

Policy information about availability of computer code

Data collection	We did not use any software for data collection.
Data analysis	Our code is made publicly available here: https://github.com/loicchadoutaud/sCellST<br>Only open source code was used for this study. All dependencies are listed in the github, and the software articles are referenced in the article.

For manuscripts utilizing custom algorithms or software that are central to the research but not yet described in published literature, software must be made available to editors and reviewers. We strongly encourage code deposition in a community repository (e.g. GitHub). See the Nature Portfolio guidelines for submitting code & software for further information.

Data

Policy information about availability of data

All manuscripts must include a data availability statement. This statement should provide the following information, where applicable:

- Accession codes, unique identifiers, or web links for publicly available datasets
- A description of any restrictions on data availability
- For clinical datasets or third party data, please ensure that the statement adheres to our policy

We accessed the spatial transcriptomic data used in this study within the HEST database [33] hosted at https://huggingface.co/datasets/MahmoodLab/hest with the provided cell segmentation. For each cancer type, we include the slide ID from HEST along with links to the original publication or source website. We excluded slides which were not preserved with FFPE and where the H&E staining quality was insufficient for the cell segmentation algorithm to perform effectively.

Visium slides HEST ids:
• Prostate: INT25, INT26, INT27, INT28, INT35
• Kidney[32]: INT13, INT14, INT15, INT17, INT18, INT19, INT21, INT24
• Breast: TENX39 (https://www.10xgenomics.com/datasets/human-breast-cancer-ductal-carcinoma-in-situ-invasive-carcinoma-ffpe-1-standard-1-3-0)
• Ovary: TENX65 (https://www.10xgenomics.com/datasets/human-ovarian-cancer-11-mm-capture-area-ffpe-2-standard)
Xenium slides HEST ids (with publication of raw dataset links):
• NCBI783, NCBI784, NCBI785 [4]
• TENX94, TENX95 https://www.10xgenomics.com/datasets/ffpe-human-breast-with-pre-designed-panel-1-standard,
• TENX96, TENX97 https://www.10xgenomics.com/datasets/ffpe-human-breast-with-custom-add-on-panel-1-standard,
• TENX98, TENX99 https://www.10xgenomics.com/datasets/ffpe-human-breast-using-the-entire-sample-area-1-standard,
single cell RNA dataset:
• Ovary: [41] https://datasets.cellxgene.cziscience.com/73fbcec3-f602-4e13-a400-a76ff91c7488. h5ad
• Breast: [43] https://datasets.cellxgene.cziscience.com/fabd4946-3f41-459c-ba79-188749a8baa4. h5ad
Source data are provided with this paper. References refer to the references in the paper.

Research involving human participants, their data, or biological material

Policy information about studies with human participants or human data. See also policy information about sex, gender (identity/presentation), and sexual orientation and race, ethnicity and racism.

Reporting on sex and gender	This is not relevant for our study, as we do not draw any conclusion at the patient level.
Reporting on race, ethnicity, or other socially relevant groupings	This is not relevant for our study, as we do not draw any conclusion at the patient level. Furthermore, as we use public data, the publication of socially relevant groupings is in the hands of the data producers.
Population characteristics	(as above)
Recruitment	(as above)
Ethics oversight	As we use public data, the publication of socially relevant groupings is in the hands of the data producers.

Note that full information on the approval of the study protocol must also be provided in the manuscript.

Field-specific reporting

Please select the one below that is the best fit for your research. If you are not sure, read the appropriate sections before making your selection.

[x] Life sciences   [ ] Behavioural & social sciences   [ ] Ecological, evolutionary & environmental sciences

For a reference copy of the document with all sections, see nature.com/documents/nr-reporting-summary-flat.pdf

Life sciences study design

All studies must disclose on these points even when the disclosure is negative.

Sample size	The sample sizes are given in the text.
Data exclusions	There have been some exclusions as explained in the text and in the answer to the reviewers.
Replication	We have conducted the experiments across several cancer types on slides from several patients. This was one of the main discussion points during the review process, and we therefore refer to our response to the reviewers.
Randomization	N/A
Blinding	N/A

Reporting for specific materials, systems and methods

We require information from authors about some types of materials, experimental systems and methods used in many studies. Here, indicate whether each material, system or method listed is relevant to your study. If you are not sure if a list item applies to your research, read the appropriate section before selecting a response.

Materials & experimental systems

n/a	Involved in the study
☒	☐ Antibodies
☒	☐ Eukaryotic cell lines
☒	☐ Palaeontology and archaeology
☒	☐ Animals and other organisms
☒	☐ Clinical data
☒	☐ Dual use research of concern
☒	☐ Plants

Methods

n/a	Involved in the study
☒	☐ ChIP-seq
☒	☐ Flow cytometry
☒	☐ MRI-based neuroimaging

Plants

Seed stocks	N/A
Novel plant genotypes	N/A
Authentication	N/A
